# 4-*tert*-Butyl-4′-(4-meth­oxy­phen­yl)-3′-(4-methyl­phen­yl)-1,2,3,4-tetra­hydro­spiro­[naphthalene-2,5′(4′*H*)-1,2-oxazol]-1-one

**DOI:** 10.1107/S1600536810044168

**Published:** 2010-10-31

**Authors:** Mohamed Akhazzane, Hafid Zouihri, Maria Daoudi, Abdelali Kerbal, Ghali Al Houari

**Affiliations:** a Laboratoire de Chimie Organique, Faculté des Sciences Dhar el Mahraz, Université Sidi Mohammed Ben Abdellah, Fès, Morocco; b Laboratoire de Diffraction des Rayons X, Centre National pour la Recherche Scientifique et Technique, Rabat, Morocco

## Abstract

In the title compound, C_30_H_31_NO_3_, the tolyl ring is almost coplanar with the isoxazole ring [dihedral angle = 12.51 (7)°], whereas the meth­oxy­phenyl ring is almost perpendicular to the isoxazole ring [dihedral angle = 89.77 (5)°]. In the crystal, mol­ecules are connected through C—H⋯O hydrogen bonds, forming chains running along the *a* axis.

## Related literature

For general background on the chemical synthesis, see: Al Houari *et al.* (2010 ▶); Bruche & Zecchi (1983 ▶); Toth *et al.* (1999 ▶).
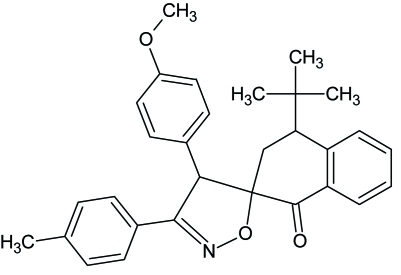


## Experimental

### 

#### Crystal data

C_30_H_31_NO_3_
*M* *_r_* = 453.56
Monoclinic, 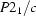

*a* = 6.9248 (3) Å
*b* = 24.7919 (12) Å
*c* = 14.2111 (7) Å
β = 94.460 (2)°
*V* = 2432.4 (2) Å^3^
*Z* = 4
Mo *K*α radiation
μ = 0.08 mm^−1^
*T* = 296 K
0.34 × 0.21 × 0.20 mm

#### Data collection

Bruker APEXII CCD detector diffractometer
21101 measured reflections
4382 independent reflections
3165 reflections with *I* > 2σ(*I*)
*R*_int_ = 0.036

#### Refinement

*R*[*F* ^2^ > 2σ(*F* ^2^)] = 0.042
*wR*(*F* ^2^) = 0.113
*S* = 1.04
4382 reflections
312 parameters
H-atom parameters constrained
Δρ_max_ = 0.14 e Å^−3^
Δρ_min_ = −0.17 e Å^−3^

### 

Data collection: *APEX2* (Bruker, 2005 ▶); cell refinement: *APEX2*; data reduction: *APEX2*; program(s) used to solve structure: *SHELXS97* (Sheldrick, 2008 ▶); program(s) used to refine structure: *SHELXL97* (Sheldrick, 2008 ▶); molecular graphics: *PLATON* (Spek, 2009 ▶); software used to prepare material for publication: *publCIF* (Westrip, 2010 ▶).

## Supplementary Material

Crystal structure: contains datablocks I, global, New_Global_Publ_Block. DOI: 10.1107/S1600536810044168/bt5399sup1.cif

Structure factors: contains datablocks I. DOI: 10.1107/S1600536810044168/bt5399Isup2.hkl

Additional supplementary materials:  crystallographic information; 3D view; checkCIF report

## Figures and Tables

**Table 1:** Hydrogen-bond geometry (Å, °)

*D*—H⋯*A*	*D*—H	H⋯*A*	*D*⋯*A*	*D*—H⋯*A*
C17—H17⋯O4^i^	0.93	2.44	3.313 (2)	156

Symmetry code: (i) 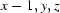.

**Fig. 1.**
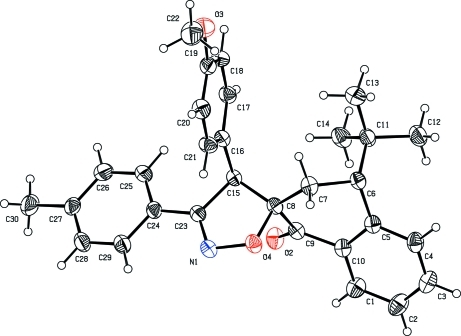
Perspective view of the title compound showing the atom-labelling scheme and 30% probability displacement ellipsoids.

**Fig. 2.**
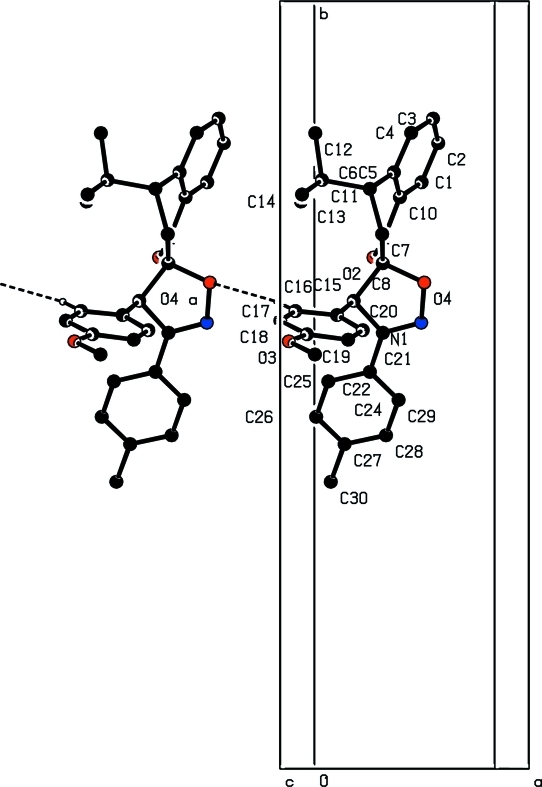
: Partial packing diagram showing two molecules connected by a C-H···O hydrogen bond.

## supplementary crystallographic information

### Comment

In the context of our research concerning the approach of dipole-dipolarophile in 1,3-dipolarcycloaddition, we have already studied the case where the dipole is an arylnitriloxide and the dipolarophiles are the 2-arylidenes of the 3,4-dihydronaphthalen-1-one substituted by anisopropyle group in position 4 (Al Houari *et al.*, 2010).

We have shown that the ring closure reaction is highly regioselective and also highly diastereoselective. The relative configuration and conformation of the products have been determined by means of protonic magnetic resonance measurements.

In this paper we describe the regiochemistry and stereochemistry in the reaction of the para-tolylnitriloxide with the 4-*tert*-butyl-2-(4-methoxybenzylidene)-3,4-dihydronaphthalen-1-one.

In general, the majority or unique regiochemistry we observe in the 1,3-dipolarcycloaddition of arylnitriloxydes with ethylenic dipolarophiles leads to anisoxazoline, where the electron-attracting  or withdrawing substitutent of the dipolarophile is in position 5 of the isoxazoline (Bruche & Zecchi 1983). This is exactly what we observed in our case with this X-ray crystal structure study, where the carbonyl group is in position 5 of the isoxazoline. We also found out, that the axial disposition the tert-butyl group imposes an exclusive anti approach of the dipole. This stereochemistry is due to steric effects.

The dihedral angles between the benzene ring of the naphthalenone and the two rings of the methylbenzene and the methoxybenzene are 58.79 (9)° and 85.36 (9)°, respctively. In the crystal, molecules are connected through C—H···O hydrogen bonds, forming chains running along the *a* axis.

### Experimental

In a 100 ml flask, we dissolved 2 mmol of the 4-*tert*-butyl-2-(4-methoxybenzylidene)-3,4-dihydronaphthalen-1-one and 2.4 mmol of *para* tolyle oxime in 20 ml chloroform. The mixture was cooled to 0°C under magnetic stirring in an ice bath. Then 15 ml of bleach at 18°C was added in small doses without exceeding 5°C. The mixture was left under magnetic stirring for 16 h at room temperature, then washed with water until the pH was neutral and dried on sodium sulfate. The solvent was evaporated with a rotating evaporator and the oily residue was dissolved in ethanol. The precipitated product was then recrystallized in ethanol.

### Refinement

All H atoms were  geometrically positioned and treated as riding with C—H ranging from  0.93 Å to 0.97Å  and with *U*_iso_(H) = 1.2Ueq(C) or *U*_iso_(H) = 1.5Ueq(C_methyl_).

### Crystal data

**Table:**

C_30_H_31_NO_3_	*F*(000) = 968
*M**_r_* = 453.56	*D*_x_ = 1.239 Mg m^−^^3^
Monoclinic, *P*2_1_/*c*	Mo *K*α radiation, λ = 0.71073 Å
Hall symbol: -P 2ybc	Cell parameters from 2572 reflections
*a* = 6.9248 (3) Å	θ = 1.7–25.1°
*b* = 24.7919 (12) Å	µ = 0.08 mm^−^^1^
*c* = 14.2111 (7) Å	*T* = 296 K
β = 94.460 (2)°	Block, yellow
*V* = 2432.4 (2) Å^3^	0.34 × 0.21 × 0.20 mm
*Z* = 4	

### Data collection

**Table:**

Bruker APEXII CCD detector diffractometer	3165 reflections with *I* > 2σ(*I*)
Radiation source: fine-focus sealed tube	*R*_int_ = 0.036
graphite	θ_max_ = 25.2°, θ_min_ = 2.9°
ω and φ scans	*h* = −7→8
21101 measured reflections	*k* = −29→29
4382 independent reflections	*l* = −17→16

### Refinement

**Table:**

Refinement on *F*^2^	Primary atom site location: structure-invariant direct methods
Least-squares matrix: full	Secondary atom site location: difference Fourier map
*R*[*F*^2^ > 2σ(*F*^2^)] = 0.042	Hydrogen site location: inferred from neighbouring sites
*wR*(*F*^2^) = 0.113	H-atom parameters constrained
*S* = 1.04	*w* = 1/[σ^2^(*F*_o_^2^) + (0.0578*P*)^2^ + 0.2609*P*] where *P* = (*F*_o_^2^ + 2*F*_c_^2^)/3
4382 reflections	(Δ/σ)_max_ = 0.005
312 parameters	Δρ_max_ = 0.14 e Å^−^^3^
0 restraints	Δρ_min_ = −0.16 e Å^−^^3^

### Special details

**Table:**

Geometry. All e.s.d.'s (except the e.s.d. in the dihedral angle between two l.s. planes) are estimated using the full covariance matrix. The cell e.s.d.'s are taken into account individually in the estimation of e.s.d.'s in distances, angles and torsion angles; correlations between e.s.d.'s in cell parameters are only used when they are defined by crystal symmetry. An approximate (isotropic) treatment of cell e.s.d.'s is used for estimating e.s.d.'s involving l.s. planes.
Refinement. Refinement of *F*^2^ against ALL reflections. The weighted *R*-factor *wR* and goodness of fit *S* are based on *F*^2^, conventional *R*-factors *R* are based on *F*, with *F* set to zero for negative *F*^2^. The threshold expression of *F*^2^ > σ(*F*^2^) is used only for calculating *R*-factors(gt) *etc*. and is not relevant to the choice of reflections for refinement. *R*-factors based on *F*^2^ are statistically about twice as large as those based on *F*, and *R*- factors based on ALL data will be even larger.

### Fractional atomic coordinates and isotropic or equivalent isotropic displacement parameters (Å^2^)

**Table:**

	*x*	*y*	*z*	*U*_iso_*/*U*_eq_	
O4	0.63085 (15)	0.63345 (4)	0.73883 (8)	0.0474 (3)	
N1	0.60997 (19)	0.58121 (5)	0.69829 (9)	0.0468 (3)	
O2	0.36687 (18)	0.66608 (4)	0.57094 (7)	0.0543 (3)	
C8	0.4373 (2)	0.65875 (6)	0.73832 (10)	0.0389 (4)	
C15	0.3017 (2)	0.60949 (6)	0.73542 (10)	0.0391 (4)	
H15	0.1879	0.6164	0.6917	0.047*	
C10	0.5015 (2)	0.74455 (6)	0.64473 (11)	0.0427 (4)	
C24	0.3633 (2)	0.51695 (6)	0.65106 (11)	0.0433 (4)	
C5	0.4863 (2)	0.77684 (6)	0.72404 (11)	0.0427 (4)	
C23	0.4304 (2)	0.56809 (6)	0.69348 (10)	0.0402 (4)	
C9	0.4249 (2)	0.68879 (6)	0.64352 (11)	0.0412 (4)	
C16	0.2379 (2)	0.59128 (6)	0.82979 (11)	0.0407 (4)	
C21	0.3692 (2)	0.57148 (7)	0.89982 (12)	0.0488 (4)	
H21	0.4973	0.5663	0.8865	0.059*	
C6	0.3864 (2)	0.75536 (6)	0.80660 (11)	0.0426 (4)	
H6	0.4421	0.7754	0.8616	0.051*	
C7	0.4479 (2)	0.69652 (6)	0.82392 (11)	0.0454 (4)	
H7A	0.5803	0.6965	0.8518	0.055*	
H7B	0.3675	0.6814	0.8703	0.055*	
C27	0.2301 (3)	0.42282 (7)	0.55619 (12)	0.0537 (5)	
O3	0.0559 (2)	0.55683 (6)	1.09572 (10)	0.0760 (4)	
C29	0.4913 (3)	0.48045 (7)	0.61566 (12)	0.0508 (4)	
H29	0.6238	0.4872	0.6233	0.061*	
C18	−0.0083 (3)	0.58377 (7)	0.93966 (14)	0.0563 (5)	
H18	−0.1374	0.5873	0.9524	0.068*	
C17	0.0469 (2)	0.59639 (7)	0.85111 (12)	0.0485 (4)	
H17	−0.0456	0.6085	0.8050	0.058*	
C4	0.5666 (2)	0.82844 (7)	0.72359 (13)	0.0524 (4)	
H4	0.5588	0.8507	0.7759	0.063*	
C20	0.3150 (3)	0.55912 (7)	0.98906 (12)	0.0545 (5)	
H20	0.4061	0.5462	1.0351	0.065*	
C1	0.5907 (3)	0.76397 (7)	0.56714 (12)	0.0554 (5)	
H1	0.5977	0.7423	0.5140	0.067*	
C28	0.4250 (3)	0.43438 (7)	0.56943 (12)	0.0573 (5)	
H28	0.5139	0.4105	0.5466	0.069*	
C14	0.0526 (2)	0.73897 (8)	0.71858 (14)	0.0625 (5)	
H14A	0.0694	0.7006	0.7240	0.094*	
H14B	0.1011	0.7512	0.6608	0.094*	
H14C	−0.0826	0.7476	0.7184	0.094*	
C11	0.1637 (2)	0.76677 (7)	0.80208 (12)	0.0493 (4)	
C19	0.1257 (3)	0.56609 (7)	1.00910 (13)	0.0528 (4)	
C25	0.1675 (3)	0.50474 (7)	0.63929 (13)	0.0589 (5)	
H25	0.0780	0.5281	0.6630	0.071*	
C26	0.1035 (3)	0.45842 (8)	0.59279 (14)	0.0654 (5)	
H26	−0.0286	0.4511	0.5861	0.078*	
C2	0.6686 (3)	0.81497 (8)	0.56862 (14)	0.0660 (5)	
H2	0.7288	0.8278	0.5168	0.079*	
C12	0.1311 (3)	0.82777 (7)	0.79301 (15)	0.0679 (6)	
H12A	−0.0038	0.8356	0.7967	0.102*	
H12B	0.1712	0.8399	0.7334	0.102*	
H12C	0.2056	0.8459	0.8433	0.102*	
C3	0.6571 (3)	0.84692 (8)	0.64697 (14)	0.0625 (5)	
H3	0.7109	0.8813	0.6483	0.075*	
C13	0.0841 (3)	0.74815 (8)	0.89371 (15)	0.0709 (6)	
H13A	−0.0504	0.7576	0.8931	0.106*	
H13B	0.1548	0.7653	0.9462	0.106*	
H13C	0.0978	0.7097	0.8995	0.106*	
C22	0.1889 (4)	0.53982 (9)	1.17053 (14)	0.0829 (7)	
H22A	0.2438	0.5057	1.1548	0.124*	
H22B	0.1229	0.5361	1.2271	0.124*	
H22C	0.2903	0.5661	1.1804	0.124*	
C30	0.1581 (4)	0.37406 (8)	0.50084 (15)	0.0777 (6)	
H30A	0.2000	0.3760	0.4381	0.117*	
H30B	0.0192	0.3731	0.4978	0.117*	
H30C	0.2093	0.3420	0.5314	0.117*	

### Atomic displacement parameters (Å^2^)

**Table:**

	*U*^11^	*U*^22^	*U*^33^	*U*^12^	*U*^13^	*U*^23^
O4	0.0394 (6)	0.0439 (7)	0.0576 (7)	0.0060 (5)	−0.0044 (5)	−0.0047 (5)
N1	0.0483 (8)	0.0428 (8)	0.0492 (8)	0.0071 (6)	0.0034 (6)	−0.0030 (6)
O2	0.0736 (8)	0.0494 (7)	0.0383 (6)	−0.0022 (6)	−0.0061 (6)	−0.0056 (5)
C8	0.0365 (8)	0.0413 (9)	0.0381 (8)	0.0063 (6)	−0.0033 (7)	−0.0006 (7)
C15	0.0378 (8)	0.0398 (9)	0.0388 (9)	0.0063 (6)	−0.0030 (7)	−0.0009 (7)
C10	0.0430 (9)	0.0430 (9)	0.0406 (9)	0.0019 (7)	−0.0069 (7)	0.0018 (7)
C24	0.0560 (10)	0.0369 (9)	0.0376 (9)	0.0032 (7)	0.0085 (8)	0.0032 (7)
C5	0.0381 (8)	0.0422 (9)	0.0460 (9)	0.0035 (7)	−0.0081 (7)	−0.0007 (7)
C23	0.0456 (9)	0.0404 (9)	0.0345 (8)	0.0062 (7)	0.0032 (7)	0.0040 (7)
C9	0.0411 (9)	0.0445 (9)	0.0372 (9)	0.0054 (7)	−0.0017 (7)	−0.0029 (7)
C16	0.0403 (9)	0.0374 (9)	0.0440 (9)	0.0038 (7)	0.0012 (7)	−0.0030 (7)
C21	0.0418 (9)	0.0546 (10)	0.0501 (10)	0.0086 (8)	0.0044 (8)	0.0047 (8)
C6	0.0453 (9)	0.0415 (9)	0.0399 (9)	0.0012 (7)	−0.0045 (7)	−0.0081 (7)
C7	0.0496 (9)	0.0461 (9)	0.0390 (9)	0.0032 (7)	−0.0068 (7)	−0.0018 (7)
C27	0.0784 (13)	0.0405 (10)	0.0451 (10)	−0.0084 (9)	0.0234 (9)	0.0001 (8)
O3	0.0921 (11)	0.0759 (9)	0.0641 (9)	0.0008 (8)	0.0322 (8)	0.0040 (7)
C29	0.0555 (10)	0.0463 (10)	0.0508 (10)	0.0090 (8)	0.0048 (8)	0.0003 (8)
C18	0.0468 (10)	0.0543 (11)	0.0704 (13)	−0.0006 (8)	0.0201 (9)	−0.0068 (10)
C17	0.0394 (9)	0.0475 (10)	0.0582 (11)	0.0040 (7)	0.0007 (8)	−0.0045 (8)
C4	0.0535 (10)	0.0447 (10)	0.0573 (11)	−0.0014 (8)	−0.0068 (9)	−0.0052 (8)
C20	0.0618 (12)	0.0540 (11)	0.0468 (10)	0.0065 (9)	−0.0008 (9)	0.0046 (8)
C1	0.0668 (12)	0.0579 (11)	0.0406 (9)	−0.0050 (9)	−0.0020 (9)	0.0042 (8)
C28	0.0785 (14)	0.0421 (10)	0.0531 (11)	0.0106 (9)	0.0167 (10)	−0.0024 (8)
C14	0.0417 (10)	0.0650 (12)	0.0790 (13)	0.0087 (8)	−0.0065 (9)	−0.0132 (10)
C11	0.0458 (9)	0.0427 (9)	0.0593 (11)	0.0038 (7)	0.0041 (8)	−0.0080 (8)
C19	0.0651 (12)	0.0439 (10)	0.0515 (10)	−0.0020 (8)	0.0183 (9)	−0.0034 (8)
C25	0.0593 (12)	0.0506 (11)	0.0696 (12)	−0.0037 (9)	0.0233 (10)	−0.0149 (9)
C26	0.0650 (12)	0.0598 (12)	0.0744 (13)	−0.0160 (10)	0.0246 (10)	−0.0136 (10)
C2	0.0793 (14)	0.0640 (13)	0.0540 (12)	−0.0154 (10)	0.0002 (10)	0.0130 (10)
C12	0.0574 (11)	0.0515 (11)	0.0953 (16)	0.0108 (9)	0.0082 (11)	−0.0084 (11)
C3	0.0661 (12)	0.0481 (11)	0.0714 (13)	−0.0119 (9)	−0.0069 (10)	0.0096 (10)
C13	0.0679 (13)	0.0667 (13)	0.0807 (15)	0.0000 (10)	0.0229 (11)	−0.0093 (11)
C22	0.123 (2)	0.0746 (15)	0.0527 (12)	−0.0013 (13)	0.0153 (13)	0.0083 (11)
C30	0.1067 (17)	0.0560 (12)	0.0748 (14)	−0.0238 (11)	0.0348 (12)	−0.0173 (11)

### Geometric parameters (Å, °)

**Table:**

O4—N1	1.4203 (16)	C18—C17	1.379 (2)
O4—C8	1.4795 (17)	C18—H18	0.9300
N1—C23	1.282 (2)	C17—H17	0.9300
O2—C9	1.2157 (17)	C4—C3	1.376 (3)
C8—C7	1.532 (2)	C4—H4	0.9300
C8—C9	1.536 (2)	C20—C19	1.374 (3)
C8—C15	1.539 (2)	C20—H20	0.9300
C15—C23	1.512 (2)	C1—C2	1.374 (3)
C15—C16	1.513 (2)	C1—H1	0.9300
C15—H15	0.9800	C28—H28	0.9300
C10—C1	1.391 (2)	C14—C11	1.527 (2)
C10—C5	1.393 (2)	C14—H14A	0.9600
C10—C9	1.480 (2)	C14—H14B	0.9600
C24—C25	1.386 (2)	C14—H14C	0.9600
C24—C29	1.388 (2)	C11—C13	1.524 (3)
C24—C23	1.464 (2)	C11—C12	1.533 (2)
C5—C4	1.395 (2)	C25—C26	1.381 (2)
C5—C6	1.505 (2)	C25—H25	0.9300
C16—C21	1.385 (2)	C26—H26	0.9300
C16—C17	1.386 (2)	C2—C3	1.374 (3)
C21—C20	1.385 (2)	C2—H2	0.9300
C21—H21	0.9300	C12—H12A	0.9600
C6—C7	1.534 (2)	C12—H12B	0.9600
C6—C11	1.565 (2)	C12—H12C	0.9600
C6—H6	0.9800	C3—H3	0.9300
C7—H7A	0.9700	C13—H13A	0.9600
C7—H7B	0.9700	C13—H13B	0.9600
C27—C26	1.375 (3)	C13—H13C	0.9600
C27—C28	1.378 (3)	C22—H22A	0.9600
C27—C30	1.506 (3)	C22—H22B	0.9600
O3—C19	1.376 (2)	C22—H22C	0.9600
O3—C22	1.416 (3)	C30—H30A	0.9600
C29—C28	1.379 (2)	C30—H30B	0.9600
C29—H29	0.9300	C30—H30C	0.9600
C18—C19	1.373 (3)		
			
N1—O4—C8	108.72 (10)	C5—C4—H4	119.5
C23—N1—O4	108.71 (12)	C19—C20—C21	119.53 (16)
O4—C8—C7	105.51 (11)	C19—C20—H20	120.2
O4—C8—C9	101.45 (11)	C21—C20—H20	120.2
C7—C8—C9	113.32 (13)	C2—C1—C10	120.28 (17)
O4—C8—C15	102.37 (11)	C2—C1—H1	119.9
C7—C8—C15	119.86 (13)	C10—C1—H1	119.9
C9—C8—C15	111.66 (12)	C27—C28—C29	121.60 (17)
C23—C15—C16	111.63 (12)	C27—C28—H28	119.2
C23—C15—C8	99.94 (12)	C29—C28—H28	119.2
C16—C15—C8	115.64 (12)	C11—C14—H14A	109.5
C23—C15—H15	109.7	C11—C14—H14B	109.5
C16—C15—H15	109.7	H14A—C14—H14B	109.5
C8—C15—H15	109.7	C11—C14—H14C	109.5
C1—C10—C5	120.57 (15)	H14A—C14—H14C	109.5
C1—C10—C9	119.66 (14)	H14B—C14—H14C	109.5
C5—C10—C9	119.77 (14)	C13—C11—C14	109.46 (16)
C25—C24—C29	117.40 (16)	C13—C11—C12	108.05 (15)
C25—C24—C23	120.98 (14)	C14—C11—C12	108.54 (15)
C29—C24—C23	121.49 (15)	C13—C11—C6	109.33 (14)
C10—C5—C4	117.91 (16)	C14—C11—C6	112.68 (13)
C10—C5—C6	119.71 (14)	C12—C11—C6	108.66 (14)
C4—C5—C6	122.38 (15)	C18—C19—C20	119.58 (16)
N1—C23—C24	121.15 (14)	C18—C19—O3	115.57 (17)
N1—C23—C15	113.93 (14)	C20—C19—O3	124.85 (18)
C24—C23—C15	124.92 (14)	C26—C25—C24	120.92 (17)
O2—C9—C10	122.47 (14)	C26—C25—H25	119.5
O2—C9—C8	120.78 (14)	C24—C25—H25	119.5
C10—C9—C8	116.56 (13)	C27—C26—C25	121.64 (19)
C21—C16—C17	117.39 (15)	C27—C26—H26	119.2
C21—C16—C15	121.45 (14)	C25—C26—H26	119.2
C17—C16—C15	121.08 (14)	C3—C2—C1	119.70 (18)
C20—C21—C16	121.81 (16)	C3—C2—H2	120.1
C20—C21—H21	119.1	C1—C2—H2	120.1
C16—C21—H21	119.1	C11—C12—H12A	109.5
C5—C6—C7	108.84 (13)	C11—C12—H12B	109.5
C5—C6—C11	114.54 (13)	H12A—C12—H12B	109.5
C7—C6—C11	116.04 (13)	C11—C12—H12C	109.5
C5—C6—H6	105.5	H12A—C12—H12C	109.5
C7—C6—H6	105.5	H12B—C12—H12C	109.5
C11—C6—H6	105.5	C2—C3—C4	120.53 (17)
C8—C7—C6	117.26 (12)	C2—C3—H3	119.7
C8—C7—H7A	108.0	C4—C3—H3	119.7
C6—C7—H7A	108.0	C11—C13—H13A	109.5
C8—C7—H7B	108.0	C11—C13—H13B	109.5
C6—C7—H7B	108.0	H13A—C13—H13B	109.5
H7A—C7—H7B	107.2	C11—C13—H13C	109.5
C26—C27—C28	117.49 (17)	H13A—C13—H13C	109.5
C26—C27—C30	121.20 (19)	H13B—C13—H13C	109.5
C28—C27—C30	121.27 (17)	O3—C22—H22A	109.5
C19—O3—C22	117.91 (17)	O3—C22—H22B	109.5
C28—C29—C24	120.93 (17)	H22A—C22—H22B	109.5
C28—C29—H29	119.5	O3—C22—H22C	109.5
C24—C29—H29	119.5	H22A—C22—H22C	109.5
C19—C18—C17	120.57 (17)	H22B—C22—H22C	109.5
C19—C18—H18	119.7	C27—C30—H30A	109.5
C17—C18—H18	119.7	C27—C30—H30B	109.5
C18—C17—C16	121.04 (16)	H30A—C30—H30B	109.5
C18—C17—H17	119.5	C27—C30—H30C	109.5
C16—C17—H17	119.5	H30A—C30—H30C	109.5
C3—C4—C5	121.00 (17)	H30B—C30—H30C	109.5
C3—C4—H4	119.5		

### Hydrogen-bond geometry (Å, °)

**Table:**

*D*—H···*A*	*D*—H	H···*A*	*D*···*A*	*D*—H···*A*
C17—H17···O4^i^	0.93	2.44	3.313 (2)	156

Symmetry codes: (i) *x*−1, *y*, *z*.
